# Using Process Evaluation Results to Compare Peer and Adult Leader Delivery of the PAWS (Peer-Education about Weight Steadiness) Club Program

**DOI:** 10.3390/nu13113901

**Published:** 2021-10-30

**Authors:** Henna Muzaffar, Sharon M. Nickols-Richardson

**Affiliations:** 1 College of Health and Human Sciences, School of Health Studies, Northern Illinois University, DeKalb, IL 60115, USA; 2 College of Agricultural, Consumer & Environmental Sciences, Food Science & Human Nutrition, University of Illinois at Urbana-Champaign, Urbana, IL 61802, USA; nickrich@illinois.edu

**Keywords:** process evaluation, program fidelity, adult leaders, peer leaders, healthy lifestyle

## Abstract

To date, there is limited published literature on process evaluation of adolescent health promotion programs. In this paper, we describe the methods and results of PAWS Club process evaluation over 2 years of implementation to compare the effectiveness of delivery by peer and adult leaders. PAWS (Peer-education About Weight Steadiness) Club was a 12-week healthy lifestyle program, delivered to 6th and 7th graders by peer and adult educators, using cluster randomized controlled design. Peer educators were 8th graders in the program schools and adult educators were staff/teachers in the program schools. Trained university students filled out fidelity logs at each session led by peer and adult educators to assess program delivery. The fidelity logs included questions to collect information about the number of participants, duration of the session, percent of activities completed, and if lessons started on time, lesson objectives were clearly stated, lesson objectives were emphasized, demonstrations were visible to participants, all activities were completed, the leader was familiar with lessons, the leader maintained an appropriate pace, the leader kept participants on track, and the leader asked if participants had any questions. Adult educators had a higher mean performance for all questions compared to peer leaders. Significant differences were observed for emphasizing lesson objectives (*p* = 0.005), making demonstrations visible to participants (*p* = 0.031), being familiar with the lesson plan (*p* = 0.000), maintaining an appropriate pace (*p* = 0.000), keeping participants on track (*p* = 0.000), and asking if participants had any questions (*p* = 0.000). Significance was set at *p* < 0.05. Findings from the current study have implications for designing and conducting a process evaluation of complex healthy lifestyle programs with adolescents in schools. Additional training of peer educators may be needed to enhance program delivery.

## 1. Introduction

Obesity in childhood and adolescence is a serious public health challenge due to its associated adverse health and social consequences [1,2]. Dietary and physical activity behaviors impact the weight status of children, and multi-component interventions targeting such behaviors can work synergistically to moderate the body weight of children and ultimately promote health [3]. Obesity prevention programs for children and adolescents present opportunities to establish healthy lifestyle behaviors that minimize the risk of gaining excess weight [4]. Studies suggest that there are fewer obesity prevention programs for adolescents than for children [5]. Recently, peer-led healthy lifestyle programs have reported positive behavior and physiologic changes among adolescents, benefitting both the peer leaders and participants [6].

The built environment and organizational, social, and communication frameworks of schools make them an ideal setting to deliver obesity prevention programs to school children and their families [6]. In the past 20 years, the afterschool environment has also been recognized as a suitable setting for delivering health promotion programs [7]. Afterschool programs are usually multi-component and target both physical activity and healthy eating and are tied to key public health recommendations [7]. Healthy lifestyle interventions have been developed and implemented in school settings with mixed results [8]. Evidence from systematic reviews of childhood obesity prevention programs suggest the unremarkable success of school-based programs [9]. Moreover, very few programs report fidelity of delivery to allow for interpretation of study findings in the context of actual delivery [10]. Program fidelity is defined as “the extent to which the intervention was delivered as it was designed or written to be delivered” [11], p. 164. Intervention fidelity results are of value to distinguish between desired outcomes not achieved due to the ineffectiveness of the program or lack of program fidelity [11,12].

Process evaluation allows researchers to assess whether interventions are delivered as intended, identify factors affecting implementation, and add context for interpretation of outcomes [5,13]. Fidelity is assessed by process evaluation of programs. The importance of process evaluation in public health interventions is increasingly recognized in research implementation science, but there is a lack of uniformity in the methods employed [14]. One of the reasons for this lack of standardized methodology is the complexity and variety of health promotion and school-based obesity prevention programs. There is even less evidence or guidance for evaluating afterschool obesity prevention programs [7,15]. Numerous frameworks have been proposed and applied for the process evaluation of health promotion programs [16,17,18,19]. To date, there is no clear guidance for combining different process evaluation factors and methods and how best to analyze collected information [15]. There is also a need to report in detail the employed process evaluation methods, as the literature suggests that reporting of such methods is poor and limited [14].

Commonly used tools for process evaluation include checklists or logbooks, interviews, focus groups, behavioral observation, and the use of administrative data including attendance or case records [13,20]. Studies suggest that the use of an external observer for process evaluation during the implementation period lends to a more valid assessment than the program staff completing the process evaluation [15]. Some of the most commonly assessed factors in process evaluation include fidelity/adherence, reach/dose/exposure, recruitment, quality, and participant responsiveness [13,15]. The United Kingdom Medical Research Council (MRC) recommends linking process evaluation results to study outcomes to better understand contextual influences, but that connection has been made in very few studies, especially for afterschool health promotion programs [7,19]. A systematic review conducted by Schaap et al. indicated that the majority of school-based obesity prevention programs did not investigate the relationship between program fidelity and program outcomes [15]. Studies linking process evaluation results with intervention outcomes can prevent misleading conclusions regarding the effectiveness of the actual intervention and inform subsequent sustainability and dissemination of intervention components [7,13,15,21,22].

### 1.1. Overview of the PAWS Club Intervention

This paper describes the results of a process evaluation undertaken to compare the fidelity of the PAWS (Peer-education About Weight Steadiness) Club intervention delivered by peer or adult educators over two years of implementation in four different schools in Eastern Illinois. The PAWS Club was a healthy lifestyle intervention implemented as an afterschool program in middle schools. The 12-week intervention focused on promoting healthy eating, physical activity, and cooking skills to prevent and reduce childhood obesity. The program was grounded in Stages of Change Learning Theory, guided by Social Cognitive Theory (SCT), and delivered by either peer or adult educators. Peer educators were 8th graders in schools, and adult educators were recruited from the staff/teachers in schools. Both peer and adult educators attended twelve sessions of training conducted by the program coordinator and practiced all teaching and hands-on activities at those sessions. The peer-led group of participants was taught by the peer educators and the adult-led group was taught by the adult educators. Outcome data were collected from participants at baseline, after the 12-week program, and 6 months later. Key outcome variables included changes in basic culinary skills, food, and physical activity behaviors, body weight, body mass index percentiles, blood pressure, and SCT mediators of behavior change [23]. In addition to evaluating program effects on the target population, process evaluation was conducted using fidelity logs tailored for each of the 12 sessions of the program.

### 1.2. Aim of the Paper

The aim of this paper is encompassed in three objectives for this study: (1) to present results of the PAWS Club process evaluation, demonstrating fidelity, quality, and completeness of delivery; (2) to compare process evaluation results between peer-led and adult-led groups; (3) to discuss PAWS Club outcome evaluation results in light of the process evaluation results.

## 2. Materials and Methods

### 2.1. Process Evaluation Methods

**Figure 1:**
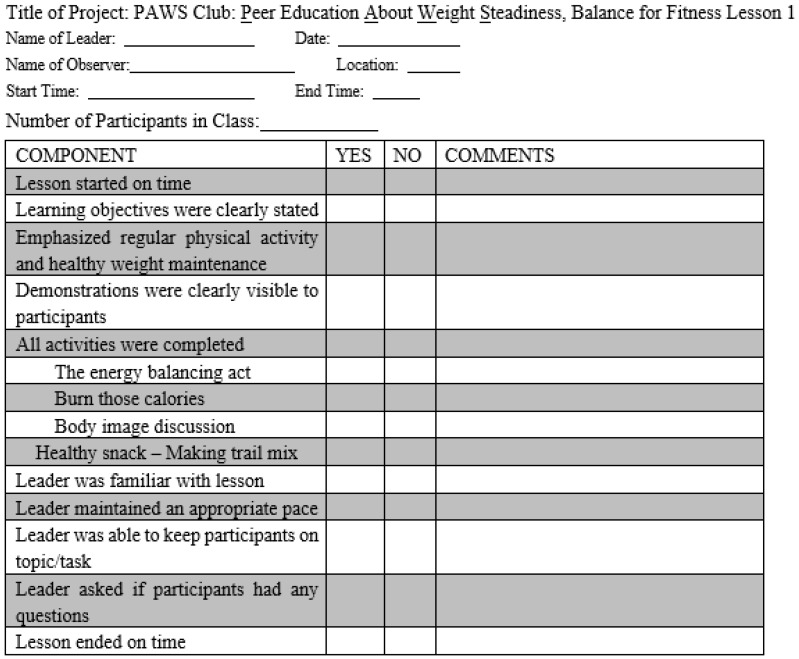
Fidelity log for lesson 1.

The PAWS Club study investigators developed separate fidelity logs for each of the 12 sessions of the program for collecting data regarding process evaluation. These logs were reviewed and assessed for face and construct validity by the PAWS Club research team. Fidelity logs were succinct and easy to comprehend to maximize completion. Each fidelity log had questions asking if: (1) Lesson started on time; (2) Lesson objectives were clearly stated; (3) Emphasized regular physical activity and healthy weight maintenance; (4) Demonstrations were clearly visible to participants; (5) All activities were completed; (6) Leader was familiar with lesson; (7) Leader maintained an appropriate pace; (8) Leader was able to keep participants on topic/task; (9) Leader asked if participants had any questions, and (10) Lesson ended on time. The response options for each of the 10 questions included yes, no, and open-ended comments [11,13]. In addition to the common questions for the 12 fidelity logs, the logs for each session contained some session-specific activities under question 5. For example, for lesson 1, titled “Balance for Fitness”, there were four additional questions: (1) The energy balance act; (2) Burn those calories; (3) Body image discussion, and (4) Healthy snack—making trail mix, with response options yes, no, and comments (Figure 1). 

University students from the nutrition, kinesiology, and human development departments at the University of Illinois at Urbana-Champaign completed a 16-week rotation with the PAWS Club program to gain experience with program delivery. They assisted with process evaluation by completing fidelity logs for each session they observed. A total of 23 university students rotated with the PAWS Club program, such that 3–4 university students engaged with the project each fall–winter and spring–summer term. Upon recruitment, the university students were given the PAWS Club manual with lesson plans and fidelity logs for all 12 sessions. To ensure inter-rater reliability, the PAWS Club research coordinator reviewed the manual with the students and trained them regarding the completion of fidelity logs for each of the sessions they observed. The university students filled out the fidelity logs throughout each wave of the 12-week PAWS Club program intervention for peer- or adult-led groups separately from 2015 to 2017. Only one fidelity log was filled out for each group of participants led by either the peer or the adult educators. After completing the 12-week rotation, university students returned completed fidelity logs to the research coordinator. Information from the fidelity logs was entered into an excel spreadsheet and checked for accuracy and completeness. If a fidelity log had four or more missing responses, those logs were excluded from data analyses (<10% of fidelity logs).

### 2.2. Details of the Fidelity Log Components

Fidelity logs were developed to assess the reach/dose (proportion of participants who received the intervention/amount of intervention delivered), fidelity (extent to which a complex intervention is implemented as intended by the developer), quality (program providers’ confidence and enthusiasm in delivering the program components), and context (aspects of the intervention setting and mode of delivery) of each of the program’s 12 sessions [16,18,24]. The PAWS Club fidelity log approach aligned with Grant et al.’s published framework for randomized controlled trials [14] and exemplified the proposed MRC guidelines [19]. 

We used structured observations and prioritized collecting quantitative data, consistent with the MRC’s recommended steps for process evaluation of a randomized controlled trial [12]. The PAWS Club process evaluators acted as observers and did not give any direct feedback to program implementers to maintain the external validity of the evaluation [25]. The reach of each lesson was assessed by two questions: number of leaders and number of participants [5]. Dose was assessed by one question: duration of lesson [5]. The fidelity of each lesson was assessed by six questions: lesson started on time; lesson objectives were clearly stated; demonstrations visible to participants; percent activities completed; leader familiar with the lesson; lesson ended on time. The quality of each lesson was assessed by four questions: emphasized objectives of lesson; leader maintained an appropriate pace; leader kept participants on track; leader asked if participants had any questions. The context of each lesson was assessed by open-ended comments recorded by university students for each of the questions on the fidelity log. University students completing fidelity logs provided both quantitative and qualitative information about the process evaluation of the PAWS Club program. For the three dose/reach questions, students noted the number of leaders, the number of participants, and the duration of the lesson in minutes. The 10 questions for session fidelity and quality had response options of yes, no, and open-ended comments. 

### 2.3. Scoring of the Fidelity Logs/Data Analyses

For quantitative data, percentages were calculated separately for peer- and adult-led sessions for each of the 10 questions with yes/no response options and means calculated for the three questions assessing dose/reach (Tables 1 and 2). A Mann–Whitney U test was conducted to assess if there were significant differences between the peer-led and adult-led sessions for the 10 program fidelity and quality questions (Table 3). A summative score was calculated for each fidelity log for peer and adult educators for all 10 yes/no response option questions to determine overall fidelity and quality of implementation [21]. For calculating the summative score, each “yes” response received a score of “1” and “no” response a score of “0”. Then, an average summative score was calculated separately for all peer educator logs and all adult educator logs to compare the difference in fidelity and quality of intervention delivery between the peer and adult educators. A Mann–Whitney U test was conducted to assess if there was a significant difference between the average summative score of peer- compared to adult-led sessions. Statistical significance was set at *p* < 0.05. For qualitative information from open-ended comments, summaries of responses for each question are compiled in Table 4 to capture the university students’ perceptions about each of the ten questions assessing fidelity and quality of program delivery. These brief summaries illustrate the impact of context on program delivery.

## 3. Results

A total of 23 university students completed fidelity logs for each of the 12 sessions of the PAWS Club program delivered to a total of 109 adolescents between 2015 and 2017. Each university student assessed program delivery by either peer or adult educators to a group of 4–6 adolescents. University students completed a total of 145 fidelity logs for peer-led sessions and 130 fidelity logs for adult-led sessions. On average, each of the 12 PAWS Club sessions was evaluated 23 times, 12 times for peer-led and 11 times for adult-led sessions congruent with the number of participant groups. Peer/adult educators, school staff, and research staff were not asked to complete fidelity logs to minimize the impact on intervention delivery. Process evaluation data are collated and enumerated in mutually exclusive tables for the peer-led and adult-led sessions (Table 1 and Table 2).

### 3.1. Process Evaluation Results

A percentage (number of yes responses/total number of responses × 100) was calculated for each evaluation indicator for each lesson and then an overall average percentage was calculated for each evaluation indicator for all 12 sessions. Process evaluation results of peer-led sessions are detailed in Table 1. The overall average scores for peer-led sessions ranged from 22 to 80%, with fidelity scores ranging from 56 to 80%, and quality scores ranging from 22 to 66%. Process evaluation results of adult-led sessions are detailed in Table 2. Overall average scores for adult-led sessions ranged from 76 to 93%, with fidelity scores ranging from 76 to 90%, and quality scores ranging from 77 to 93%. The percentage scores were stratified into quartiles for low (<25%), medium (>25–<75%), and high implementation (>75%) (Table 3), as per previous process evaluations [7,22].

### 3.2. Comparison between Peer-Led and Adult-Led Sessions

For the three questions assessing dose/reach, duration was longer for adult-led sessions, and the ratio of leaders to participants was higher for the peer-led sessions (Table 1, Table 2 and Table 3). For the six fidelity questions, there was a difference of 6–15% between the peer-led and adult-led groups (Table 1, Table 2 and Table 3). For the four quality questions, there was a difference of 22–55% between the peer-led vs. adult-led groups (Table 1, Table 2 and Table 3). Adult educators had a higher mean performance for all 10 fidelity and quality questions compared to peer educators. The Mann–Whitney U test results indicated significant differences between adult and peer leaders for emphasizing objectives of lessons (*p* = 0.005), making demonstrations visible to participants (*p* = 0.031), being familiar with the lesson plan (*p* = 0.000), maintaining an appropriate pace (*p* = 0.000), keeping participants on track (*p* = 0.000), and asking if participants had any questions (*p* = 0.000). Thus, all four quality scores and two fidelity scores were significantly different between the peer- vs. adult-led sessions. For the summative score, the average level of implementation for the peer-led sessions was 6.26 (medium) and adult-led sessions was 8.34 (high). For previous studies calculating a summative score, 8 and above was characterized as a high level of implementation and 6 as medium level [11,13]. Mann–Whitney U test results indicated a significant difference between the summative score for peer-led vs. adult-led sessions (*p* = 0.000).

University students’ notations on fidelity logs supported the quantitative results of process evaluation. For all six questions with significant differences between peer and adult educators, qualitative comments provided additional information about the contrast in program delivery by peer and adult educators (Table 4). These comments also highlight the contextual differences between the peer-led and adult-led sessions and how those differences may have impacted the delivery of the program.

## 4. Discussion

In this paper, we have presented in detail the methods and results of process evaluation conducted throughout PAWS Club program implementation. Overall, the program achieved medium to high implementation, and 80% or more of the program activities were completed in both peer-led and adult-led sessions. The adult-led sessions scored higher for all 10 fidelity and quality evaluation indicators as compared to peer-led sessions. Significant differences were observed between the peer- and adult-led groups for all four quality indicators and two fidelity indicators. Another noteworthy finding was that for adult-led sessions, all 10 fidelity and quality indicators scored in the high implementation range; whereas for peer-led sessions, two were in the high category, seven in the medium range, and one in the low implementation category. Lastly, the summative score for adult-led sessions was in the high implementation range and for peer-led sessions in the medium implementation range. 

Previously published process evaluation studies suggest that training of program leaders, ongoing support from the research team, and access to a program manual promote compliance with program delivery plans [11,22,26,27]. We attribute the moderate/high intervention fidelity of the PAWS Club program to the peer and adult leaders receiving 12 sessions of training focused on program content and activities, provision of program manuals with clear descriptions of session objectives and activities, and ongoing support from the research staff at each session. However, there was a greater discrepancy in the quality measures between the peer-led and adult-led sessions. Training for peer and adult leaders did not include strategies for handling group instruction, dealing with behavioral issues, engaging participants in a session, or time management skills which have been shown to improve the implementation level of interventions [6]. Teachers possess these skills as they practice these attributes of instruction on a daily basis and can extrapolate them to similar situations [28]. Peer educators in schools could have benefitted from such training, which would likely have improved the quality of peer-led PAWS Club program sessions.

Many previous childhood obesity prevention trials have either not conducted comprehensive evaluations of program implementation or have not reported methods and results of process evaluation in detail [13]. Even though there is increasing evidence of the importance of undertaking process evaluation, there is no established protocol on how this should be done. Since 2008, the MRC has been developing and updating guidance for process evaluation of complex health promotion programs, and their frameworks have informed process evaluation of health promotion programs [12,29]. The MRC’s latest guidance framework published in 2015 serves as an evidence-based skeleton plan. The MRC recognizes that more program fidelity evaluation efforts need to be reported to finalize recommendations, as there remains a paucity of studies publishing their process evaluation efforts.

The MRC has delineated three core aims of process evaluation: (1) evaluation of quality and quantity of program implementation; (2) explanation of theoretical connections between the program delivered and its intended effects, and (3) assessment of the impact of contextual factors on the delivery and outcomes of the program [12]. Within the constraints of our study design, we have addressed all three core aims of process evaluation in our efforts for PAWS Club implementation evaluation. Combining process evaluation results with study outcomes tests the theoretical pathways of change and enhances the interpretation of findings [12]. Such efforts are being increasingly emphasized for researchers to plan from the beginning and not as an afterthought once the program is delivered [30]. Examining intervention implementation separately for peer-led and adult-led sessions has enabled us to assess how well the intervention was delivered in peer- vs. adult-led sessions and also conduct exploratory analyses to assess any associations between study outcomes and the degree of fidelity and the level of quality achieved in program implementation. However, we could not apply any statistical models to investigate the influence of intervention fidelity and quality on study outcomes, as we did not have individual-level data for process evaluation to match outcome evaluation data.

Our study results indicate that adult-led sessions scored higher than the peer-led sessions for all dimensions of fidelity and quality, with more significant differences with peer leaders for the quality constructs. Results of our study underscore the importance of conducting comparative process evaluation for both peer-led and adult-led groups to allow for interpretation of the intervention outcomes and for identifying best practices and barriers to implementation of such programs. Findings from the PAWS Club outcome effectiveness trial indicated that both the peer-led and adult-led groups showed significant changes in dietary intake by reducing caloric intake [23]. The medium to high fidelity of PAWS Club program delivery for both groups could have possibly led to this positive dietary change. A separate study linking process evaluation results with study outcomes also indicated similar improvement in healthy eating outcomes for both high and low implementation groups, but physical activity outcomes improved more in the high implementation group [7]. Furthermore, the PAWS Club adult-led group showed improvement in the self-regulation and outcome expectations constructs of SCT [23]. The high quality of program delivery by adult educators has a positive association with this significant improvement in psychosocial variables, self-regulation, and outcome expectations, in the adult-led group. Another significant outcome of the PAWS Club program was that the peer-led group increased their intake of whole grains [23]. The increased intake of whole grains by the peer-led group suggests that there was a positive influence of peers when encouraging unfamiliar food items. Lastly, the PAWS Club participants from both peer-led and adult-led groups showed positive but non-significant improvements for family mealtime frequency, fat intake, salt intake, and sugar intake [23]. The medium to high fidelity of the PAWS Club program delivery for both peer- and adult-led sessions may have contributed to these additional positive outcomes. These are all suggested associations and more comprehensive health promotion programs are needed to substantiate our results and guide the science forward in identifying best practices for increasing the effectiveness of similar obesity prevention efforts.

### 4.1. Strengths

We collected process evaluation data for all 12 sessions of the PAWS Club program, for each wave of implementation from 2015 to 2017, for both peer-led and adult-led sessions in all four schools. Thus, a large sample of fidelity logs was available for analyses and inferences [31]. To improve inter-rater reliability in process evaluation, university students received a detailed program manual, training, and fidelity checklists to assess program reach, dose, fidelity, and quality. University trainees filled out both qualitative and quantitative information on the checklist, providing a more comprehensive picture of intervention integrity. Use of a cumulative score for all fidelity and quality constructs and the stratification of each fidelity/quality construct to high/medium/low level of implementation allowed for a more thorough evaluation of the level of intervention fidelity and quality achieved by the peer and adult educators. Lastly, university students assessed adherence to the PAWS Club intervention as independent observers, not delegated to program delivery thereby increasing the validity of findings [15].

### 4.2. Limitations

The validity and reliability of fidelity logs were not assessed fully, as there is no current gold standard tool for fidelity assessment and most tools are intervention specific. However, we did establish face and construct validity of the log through confirmation with experts. Secondly, the peer and adult educators were aware that the university students were observing and completing implementation evaluation forms during the session, which may have influenced the delivery of sessions. However, the fidelity log results suggest that this did not happen, as the educators were not privy to any details on the fidelity log and the university students did not give any direct feedback to the educators. Thirdly, we used a single instrument, fidelity logs, to assess program fidelity and quality, which does not allow for data triangulation to confirm the findings. However, there was congruence between quantitative and qualitative data collected via fidelity logs. Process evaluation using high quality and multiple resources may not always be practical in real-world settings, as researchers need to balance the quality of measures used for process evaluation with participant and researcher burden [15]. Lastly, we did not collect demographic and outcome evaluation data from peer educators to compare them with the participants.

## 5. Conclusions

Methods detailed in this paper will add to the process evaluation literature. The PAWS Club process evaluation strategy builds on the methods and findings of comparable interventions and assists with refining the methods and frameworks for future process evaluation efforts. Our study results indicate that both peer and adult leaders delivered the program with medium to high fidelity, but the quality of intervention delivered by adult leaders was significantly better than by peer leaders. As the program fidelity was maintained for both groups, the study effectiveness findings are a reflection of the program’s potential for positive changes. Moreover, our work highlights the importance of assessing the quality of program delivery, as some study effectiveness outcomes might have been influenced by the quality of intervention delivered. Future studies can assess if the quality and fidelity of programs can be improved by providing additional training to peer educators, providing more detailed lessons to peer educators, providing ongoing training, and allowing peer and adult educators to deliver the program together. To fill gaps in the literature, more studies need to be published to build the evidence base for valid, reliable, high quality, and feasible process evaluation tools and methods that can be used for obesity prevention programs. Such work will elucidate the relationship between program fidelity and program outcomes, which will aid in understanding the level of fidelity required for programs to be effective. Lastly, researchers need to adopt a multifaceted approach, encompassing outcome and process evaluation, to comprehensively evaluate complex obesity prevention programs. 

## Figures and Tables

**Table 1:** Process evaluation (dose (D), reach (R), fidelity (F), quality (Q)). Results of peer-led sessions.

Sessions	1	2	3	4	5	6	7	8	9	10	11	12	Ave
Number of lessons evaluated	13	12	13	12	13	12	12	12	12	10	12	12	12
Number of participants (R)	6	6	6	4	4	5	4	4	4	4	5	4	5
Number of leaders (R)	3	3	2	2	3	3	3	3	3	3	3	3	3
Duration (minutes) (D)	64	71	66	70	71	70	75	69	62	60	70	63	68
Lesson started on time (%) (F)	46	100	46	92	62	83	75	66	66	50	75	92	71
Lesson objectives were clearly stated (%) (F)	77	50	62	33	38	75	92	33	75	50	75	75	61
Emphasized objectives of the lesson (%) (Q)	85	83	46	33	62	58	83	42	66	40	75	75	62
Demonstrations visible to participants (%) (F)	69	100	92	66	85	83	92	58	83	70	83	75	80
% Of activitiesActivities completed (F)	98	88	78	92	80	72	85	55	82	67	81	77	80
Leader familiar with lesson (%) (F)	62	42	46	83	46	50	42	58	66	50	66	58	56
Leader maintained an appropriate pace (%) (Q)	69	83	77	92	38	50	58	75	66	50	58	75	66
Leader kept participants on track (%) (Q)	69	50	77	58	54	75	66	66	58	20	50	75	60
Leader asked if participants had any questions (%) (Q)	31	33	46	25	8	25	25	25	17	0	8	17	22
Lesson ended on time (%) (F)	54	67	54	92	85	75	75	75	75	30	75	83	70

**Table 2:** Process evaluation (dose (D), reach (R), fidelity (F), quality (Q)). Results of adult-led sessions.

Sessions	1	2	3	4	5	6	7	8	9	10	11	12	Ave
Number of lessons evaluated	8	12	9	12	9	12	10	12	10	14	9	13	11
Number of participants (R)	7	6	6	6	6	6	6	6	5	6	6	5	6
Number of leaders (R)	2	1	2	1	2	1	2	2	2	2	2	2	2
Duration (minutes) (D)	65	78	76	80	80	78	76	77	75	79	82	60	76
Lesson started on time (%) (F)	50	83	66	100	89	83	90	75	70	86	78	77	79
Lesson objectives were clearly stated (%) (F)	63	42	89	66	78	83	90	75	100	64	89	69	76
Emphasized objectives of the lesson (%) (Q)	88	83	56	58	89	92	100	83	90	86	100	77	84
Demonstrations visible to participants (%) (F)	75	92	100	92	89	92	100	83	100	86	89	77	90
% Of activitiesActivities completed (F)	100	85	95	100	91	86	93	71	92	80	68	75	86
Leader familiar with lesson (%) (F)	75	66	100	92	89	100	100	92	100	93	89	69	89
Leader maintained an appropriate pace (%) (Q)	100	92	78	92	78	100	100	83	90	93	89	77	89
Leader kept participants on track (%) (Q)	100	92	100	92	100	92	100	92	90	86	89	77	93
Leader asked if participants had any questions (%) (Q)	88	75	78	66	56	83	90	75	90	79	89	54	77

**Table 3:** Comparison of process evaluation between peer and adult educators.

Evaluation Criteria	Peer-Led Sessions	Adult-Led Sessions	Difference between Peer- and Adult-Led Sessions	*p*-Value
Number of times each lesson evaluated	12	11	1	N/A
Number of leaders (R)	2–5	1–3	1–2	N/A
Number of participants (R)	4–5	5–6	1	N/A
Duration of the lesson (D)	68 min	76 min	8 min	N/A
Lesson started on time (F)	71% (medium)	79% (high)	8%	0.241
Lesson objectives were clearly stated (F)	61% (medium)	76% (high)	15%	0.062
Emphasized objectives of lesson (Q)	62% (medium)	84% (high)	22%	0.005 *
Demonstrations visible to participants (F)	80% (high)	90% (high)	10%	0.031 *
% Of activities completed (F)	80% (high)	86% (high)	6%	0.249
Leader familiar with lesson (F)	56% (medium)	89% (high)	33%	0.000 *
Leader maintained an appropriate pace (Q)	66% (medium)	89% (high)	23%	0.000 *
Leader kept participants on track (Q)	60% (medium)	93% (high)	33%	0.000 *
Leader asked if participants had any questions (Q)	22% (low)	77% (high)	55%	0.000 *
Lesson ended on time (F)	70% (medium)	78%(high)	8%	0.205

Mann–Whitney U Test; * Significance set at *p* < 0.05; N/A = Not Applicable; F = Fidelity; Q = Quality. The percentage scores stratified into quartiles: low (<25%), medium (>25%–<75%), and high (>75%) implementation.

**Table 4:** Summary of university student comments for fidelity log questions for peer- and adult-led sessions.

Fidelity Log Question	Summary for Peer Educators	Summary for Adult Educators
Lesson started on time	Most of the lessons started a little late due to either the educators coming late, the participants coming late, time finishing up questionnaires, two lessons for the same day, peer educators not prepared well, and sometimes a snack was served first.	Lessons started a little late most of the time due to adult educators coming in late due to finishing up their work for the day, or the kids coming late, or they were served snacks a little earlier, or the participants had to finish up the study questionnaires.
Lesson objectives were clearly stated	The lesson objectives were not explicitly stated most of the time. Sometimes the peer educators read the overview or mentioned a couple of objectives. Mostly the peer educators read the directions for the activities from the lesson plans. Sometimes the peer educators asked the participants questions before starting the class.	Most of the time the lesson objectives were not clearly stated by the adult educators. They did explain what they would do in class that day or go over the overview of that day’s lesson. Sometimes the adult educators would also go over what was learned in the last session.
Emphasized objectives of lesson	The peer educators did not emphasize all the lesson objectives. Some of the lesson objectives that they discussed more were how to be more physically active and the health impacts of exercise, eating breakfast, and eating fruits and vegetables.	The adult educators emphasized physical activity, key benefits of physical activity, ways to eat different types of fruits and vegetables, healthy snacks, how Americans eat out a lot, different restaurant foods, amount of sugar in drink labels, goal setting, food labels, family mealtimes, shopping and planning for meals, and MyPlate.
Demonstrations visible to participants	Peer educators made the demos visible to the participants. Sometimes the participants were not interested because they did not understand the activity, and peer educators did not explain the purpose of the activities but sometimes asked the participants questions after activities.	Adult educators sometimes did the demos in the front of the room instead of doing it at the table to prevent crowding at the table. Adult educators gave effective personal examples and gave clear instructions and explanations for the participants to understand the activity and be able to perform the activity on their own as well.
All activities were completed	Peer educators often missed some activities, rushed through some, or went out of order.	Completed activities most of the time. Sometimes missed activities or modified them.
% Of activities completed	No comments	No comments
Leader familiar with lesson	Peer educators mostly were not familiar with the lesson, did the activities out of order, and read from the lesson plans during the session. They were interactive with participants and were good facilitators. They needed help/reminders from the site coordinator. Sometimes they did not know which lesson was assigned for the day. Some skipped parts of the lesson or forgot to bring program binders.	Adult educators most of the time were familiar with the lesson, and sometimes added a lot of extras and reviewed the lesson to make sure all the content is covered.
Leader maintained an appropriate pace	Peer educators were mostly slow in delivering the lesson and encouraged too many distractions, lingered on at snack time, needed to be told when to move on, skipped some points in the lesson, went out of order, and had gaps in the lesson because they were unprepared. Sometimes they finished the lesson quickly and without much discussion with the participants.	Adult educators maintained a good pace most of the time to ensure all the content was covered. They sometimes added extra information. They also asked the kids if they had any questions related to the content.
Leader kept participants on track	Most of the time the peer educators were not able to keep the participants on the topic because they were reading from the program binders, participants started side convo and the educators encouraged off-topic chatting, participants interrupted the lesson by distracting other children, peer leaders were on their phones, kids were rowdy and educators unable to control, and sometimes leaders lost focus and did not cover the content sufficiently. Sometimes the peer educators were able to put them back on task after the interruptions.	Adult educators did their best to keep the participants on task/topic, asked questions to keep the participants engaged, redirected and brought the kids to focus again after distractions such as snack time or physical activity. Sometimes the kids did not cooperate.
Leader asked if participants had any questions	Peer educators most of the time just lectured or read from the lesson plans and very rarely asked questions. However, if the participants asked questions, they answered them thoughtfully.	Adult educators encouraged discussion by asking questions, discussed everything more than once, reviewed the previous lesson, and answered all the questions and turned them into learning opportunities.
Lesson ended on time	Most of the time the lesson ended a little early but around the expected end time or a few times it ended too late.	Most of the time the lesson ended a little early but the adult educators gave a good review and reminders at the end.

## Data Availability

The datasets used and/or analyzed during the current study are available from the corresponding author on reasonable request.
